# TRAF5-mediated Tax ubiquitination modulates IKK phosphorylation but not binding to NEMO

**DOI:** 10.1186/1742-4690-11-S1-P102

**Published:** 2014-01-07

**Authors:** Amandine Bonnet, Sabrina Pène, Laurence Bénit, Laetitia Waast, Ali Bazarbachi, Renaud Mahieux, Claudine Pique

**Affiliations:** 1 INSERM, U1016, Institut Cochin, CNRS, UMR8104, Université Paris Descartes, Sorbonne Paris Cité, Paris, France; 2 Department of Internal Medicine, American University of Beirut, Beirut, Lebanon; 3 Oncogenèse Rétrovirale, CIRI, INSERM U1111-CNRS UMR5308, Université Lyon 1, Ecole Normale Supérieure de Lyon, LabEx ECOFECT, Lyon, Cedex 07, France

## 

Tax is a powerful activator of the NF-kB pathway, a property shown to be required for HTLV-1-induced immortalization of primary T lymphocytes. A pivotal step in the stimulation of this pathway is the activation of the cytoplasmic IkB-kinase (IKK) complex, which consists of two catalytic subunits, IKK-alpha and beta and a regulatory subunit, IKK-gamma/NEMO. Previous studies showed that the ability of Tax to bind to and activate the IKK complex depends on its prior conjugation to ubiquitin. TRAF5, a member of the TNF Receptor-Associated Factor family, is an adaptor protein and E3 ubiquitin ligase which functions downstream various membrane receptors, notably for the activation of the NF-kB pathway. Interestingly, TRAF5 was also shown to interact with the Epstein Barr Virus (EBV)-encoded LMP1 oncoprotein and to contribute to LMP1-induced IKK activation. In this study, we investigated whether TRAF5 could also be a functional partner of Tax. We found that overexpressing TRAF5 significantly increases endogenous Tax ubiquitination while conversely endogenous Tax ubiquitination is reduced upon siRNA-mediated TRAF5 silencing. Surprisingly, preventing TRAF5-mediated Tax ubiquitination by siRNA depletion of TRAF5 does not affect Tax binding to endogenous NEMO. However, Tax-induced phosphorylation of IKK-alpha/beta is significantly decreased in the same setting, which coincided with a decreased ability of Tax to activate a NF-kB promoter. These findings reveal that TRAF5 mediates Tax ubiquitination for IKK activation and suggest that Tax binding to NEMO and Tax-induced IKK phosphorylation are regulated by distinct molecular determinants.

